# Supplementary material for: Can kinesio tape negatively affect the treatment by creating a hard floor in plantar fasciitis treatment? A randomized clinical trial
Source: PLoS One. 2025 May 5;20(5):e0322397. doi: 10.1371/journal.pone.0322397 (PMC12052111; doi:10.1371/journal.pone.0322397)
Supplement: S1 Study Protocol — (DOCX) [file pone.0322397.s002.docx]

*(Kırmızı çerçeve içindeki alanlar Klinik Araştırmalar Etik Kurulu Sekretaryası tarafından doldurulacaktır*).

| Başvurunun yapıldığı tarih: [ ] |  | Red/olumsuz görüş nedenleri: [ ]  Tarih: [ ] |  |
| --- | --- | --- | --- |
| Başvuru arşiv kayıt numarası: | | |  |
| Düzeltme yapıldı ise tarihi: | | |  |

**ETİK KURUL BİLGİLERİ**

| **1.** | **Araştırma onayı için daha önce Etik Kurul başvurusu yapıldı mı?** |  |  |
| --- | --- | --- | --- |
| **1.1.** | Cevabınız evet ise: | | |
| **1.1.1.** | Etik Kurulun adı: [ ] | | |
| **1.1.2.** | Başvuru tarihi: [ ] | | |

**A. ARAŞTIRMA**

| **A.1** | **Tüm araştırmacıların adı**: [Doç.Dr. Tuğba KOCAHAN, Doç.Dr. Aydan ÖRSÇELİK, Doç.Dr. Bihter AKINOĞLU] | | | |
| --- | --- | --- | --- | --- |
| **A.2** | **Araştırmanın adı** *(Kısaltma kullanılmamalı ve araştırma amacını yansıtmalı)*: [Plantar Fasiitiste Ekstrakorporeal Şok Dalga Tedavisi (ESWT) ile Birlikte Uygulanan Kinesiotaping Uygulamalarının Etkinliğinin İncelenmesi] | | | |
| **A.3** | **Araştırma pediyatrik araştırma planının bir parçası mı?** | **** | **** | |
| **A.4** | **Araştırmanın statüsü** *(Aşağıdaki kutucuklardan uygun olanı işaretleyiniz.)* | | | |
| **A.4.1** | Yüksek lisans tezi | | |  |
| **A.4.2** | Doktora tezi | | |  |
| **A.4.3** | Uzmanlık tezi | | |  |
| **A.4.4** | Bireysel araştırma | | |  |
| **A.4.5** | Çok merkezli araştırma | | |  |
| **A.4.6** | **Diğer ise, belirtiniz:** [ ] | | | |

**B. DESTEKLEYİCİ**

| **B.1.** | **Araştırmanın destekleyicisi var mı** *(mevcut/planlanan)***?** |  |  | |
| --- | --- | --- | --- | --- |
| **B.1.1** | **B.1**’e cevabınız evet ise, aşağıdaki uygun kutucuğu işaretleyiniz. | | | |
| **B.1.1.1** | Üniversite (Bilimsel Araştırma Projeleri Koordinasyon Birimi; *BAP*) | | |  |
| **B.1.1.2** | Eğitim ve Araştırma Hastanesi | | |  |
| **B.1.1.3** | TÜBİTAK *(Türkiye Bilimsel ve Teknolojik Araştırma Kurumu)* | | |  |
| **B.1.1.4** | DPT *(Devlet Planlama Teşkilatı)* | | |  |
| **B.1.1.5** | Uluslararası ise belirtiniz: [ ] | | | |
| **B.1.1.6** | Diğer ise (özel kuruluş, vb), belirtiniz: [ ] | | | |

**C. ARAŞTIRMAYA İLİŞKİN GENEL BİLGİLER**

| **C.1** | | **Araştırılan tıbbi durum veya hastalık** | |
| --- | --- | --- | --- |
| **C.1.1** | | Araştırılan tıbbi durum veya hastalıkları belirtiniz *(serbest metin olarak belirtiniz)*: [Plantar fasiitis tanısı alan hastalar] | |
| **C.1.1.1** | | Çalışma alanını (fizyoloji, saha çalışması, onkoloji, hematoloji, vb) belirtiniz: [ Spor Hekimliği, Fizyoterapi] | |
| **C.2** | **Araştırmanın türü** (*Uygun olan kutu/kutuları işaretleyiniz.*) | | |
| **C.2.1** | Dosya ve görüntü kayıtları gibi, arşiv taramalarına dayanan retrospektif çalışmalar |  | |
| **C.2.2** | Sağlıkla ilgili anket ve benzeri veri toplama araçları ile yapılan araştırmalar |  | |
| **C.2.3** | Bilgisayar ortamında test, mülakat, ses/video kaydı ile toplanan verilerin kullanılacağı araştırmalar |  | |
| **C.2.4** | **İlaç dışı gözlemsel çalışmalar:**Tanımlayıcı  Kesitsel  Olgu-Kontrol  Kohort |  | |
| **C.2.5** | Kan, idrar, doku, saç, tüy, tükürük, gaita ve radyolojik görüntü gibi biyokimya, mikrobiyoloji, patoloji ve radyoloji materyalleri ile yapılan arştırmalar |  | |
| **C.2.6** | Rutin muayene, tahlil ve tedavi işlemleri sırasında elde edilen materyal ile yapılan çalışmalar |  | |
| **C.2.7** | Egzersiz gibi vücut fizyolojisi ile ilgili araştırmalar |  | |
| **C.2.8** | Hemşirelik faaliyetlerinin sınırları içerisinde yapılacak araştırmalar |  | |
| **C.2.9** | Hücre veya doku kültürü (*in vitro*) araştırmaları |  | |
| **C.2.10** | Gen tedavisi dışında kalan ve tanımlamaya yönelik genetik materyalle yapılacak çalışmalar |  | |
| **C.2.11** | Antropometrik ölçümlere dayalı yapılan araştırmalar, |  | |
| **C.2.12** | Beslenme gibi yaşam alışkanlıklarının değerlendirilmesi çalışmaları |  | |
| **C.2.13** | Diğer ise, belirtiniz: [İki farklı tedaviyi birlikte uygulamanın etkinliğinin incelenmesi] |  | |

| **C.3** | **Araştırmanın tasarımı** | | |
| --- | --- | --- | --- |
| **C.3.1** | Kontrollü (kontrol grubu içeren araştırma) |  |  |
| **C.3.2** | Diğer ise, belirtiniz: [ ] | | |

| **C.4** | **Araştırma merkezi** | | | |
| --- | --- | --- | --- | --- |
| **C.4.1** | Tek merkez var |  |  | |
| **C.4.2** | Birden çok merkez var |  |  | |
| **C.4.2.1** | Ülkemizde öngörülen merkez sayısı ve isimlerini belirtiniz: [ SBÜ GEAH Spor Hekimliği] | | | |
| **C.4.3** | Bu araştırma başka ülkelerde de yürütülecek mi? |  |  | |
| **C.4.3.1** | **C.4.3**’e cevabınız evet ise, başka ülkelerde öngörülen merkez sayısını ve ülkeleri belirtiniz: | | | |
| **C.4.4** | Çalışma kapsamında toplanan biyolojik materyallerin, yurt içi veya yurt dışı başka bir kuruma gönderilmesi planlanıyor mu? |  |  | |
| **C.4.4.1** | **C.4.4**’e cevabınız evet ise, açıklayınız: [ ]  [www.titck.gov.tr](http://www.titck.gov.tr) adresinde yer alan güncel biyolojik materyal transfer antlaşmasını başvuru dosyasına ekleyiniz. | | | |
| **C.5** | **Araştırma süresi** *(gün, ay ve yıl olarak):* | | | |
| **C.5.1.** | Araştırmanın ülkemizde tahminen ne kadar süreceğini belirtiniz: | [ ] | [ ] | [1 yıl ] |
| **C.5.1.2** | Varsa, araştırmada yer alan bütün ülkelerde araştırmanın tahminen ne kadar süreceğini belirtiniz: | [ ] | [ ] | [ ] |
| **C.6** | **Araştırmaya gönüllü almaya başlamak için önerilen tarih** *(gün, ay ve yıl olarak):* | | | |
| **C.6.1** | Ülkemizdeki tarihi belirtiniz: | [01] | [08] | [2023] |
| **C.6.1.2** | Varsa, diğer ülkelerdeki tarihi belirtiniz: | [ ] | [ ] | [ ] |

| **C.7** | **Araştırmada kullanılan veri toplama yöntemi** (*Uygun olan kutu/kutuları işaretleyiniz.*) | |
| --- | --- | --- |
| **C.7.1** | Anket |  |
| **C.7.2** | Mülakat |  |
| **C.7.3** | Gözlem |  |
| **C.7.4** | Dosya taraması (retrospektif) |  |
| **C.7.5** | Bilgisayar ortamında test uygulaması |  |
| **C.7.6** | Görüntü kaydı |  |
| **C.7.7** | Ses kaydı |  |
| **C.7.7.1** | Biyolojik materyal üzerinde yapılan çalışma | |
| **C.7.7.2** | *In vivo* çalışma |  |
| **C.7.7.3** | *In vitro* çalışma |  |
| **C.7. 8** | *Ex vivo* çalışma |  |
| **C.7.9** | Post-mortem |  |
| **C.7.10** | Diğer ise, belirtiniz…: [ ] | |

**D. ARAŞTIRMAYA ALINAN GÖNÜLLÜLER**

| **D.1** | **Yaş aralığı** (*Araştırmanın tamamı için her yaş aralığında planlanan tahmini gönüllü sayısını belirtiniz.*) | | |
| --- | --- | --- | --- |
| **D.1.1** | 18 yaş altı |  |  |
| **D.1.1.1** | **D.1.1**’e cevabınız evet ise, yaş aralığını ve gönüllü sayısını belirtiniz: [] | | |
| **D.1.2** | 18 yaş üstü: [ 44 ] |  |  |
| **D.1.2.1** | **D.1.2**’ye cevabınız evet ise yaş aralığını ve gönüllü sayısını belirtiniz: Başvurmuş olan tüm yaş grupları | | |

| **D.2** | **Cinsiyet** | |
| --- | --- | --- |
| **D.2.1** | Kadın |  |
| **D.2.2** | Erkek |  |

| **D.3** | **Araştırmadaki gönüllü grubu** | |
| --- | --- | --- |
| **D.3.1** | Sağlıklı gönüllüler |  |
| **D.3.2** | Hastalar |  |
| **D.3.3** | Özel hassas gruplar |  |
| **D.3.3.1** | Doğum kontrol yöntemi kullanmayan ve çocuk doğurma potansiyeli olan kadınlar |  |
| **D.3.3.2** | Doğum kontrol yöntemi kullanan ve çocuk doğurma potansiyeli olan kadınlar |  |
| **D.3.3.3** | Gebe kadınlar |  |
| **D.3.3.4** | Emziren kadınlar |  |
| **D.3.3.5** | Acil olgular |  |
| **D.3.3.6** | Şahsen olur veremeyecek gönüllüler |  |
| **D.3.3.6.1** | **D.3.3.6’**ya cevabınız evet ise, belirtiniz: [ ] | |
| **D.3.3.7** | Diğer ise, belirtiniz: [ ] | |
| **D.4** | **Gönüllülerin araştırmaya dahil edilme kriterleri** *(Maddeler halinde sıralayınız).*  *(Varsa, kontrol grubu olarak, sağlıklı gönüllüler için de ayrı sıralama yapılmalıdır).* | |
|  | 1. PF tanısı almış olmak 2. Bilinen herhangi bir sistemik problemi olmaması, 3. Alt ekstremite ile ilgili herhangi bir cerrahi operasyon geçirmemiş olması, 4. Son üç ay içerisinde alt ekstremite yaralanması geçirmemiş olması, 5. 18-65 yaş arası olmak 6. Kadın ve Erkek gönüllü olmak. | |
| **D.5** | **Gönüllülerin araştırmaya dahil edilmeme kriterleri** *(Maddeler halinde sıralayınız.)*  *(Varsa, kontrol grubu olarak, sağlıklı gönüllüler için de ayrı sıralama yapılmalıdır.)* | |
|  | 1. Gebelik, 2. Kanama bozukluğu, 3. Kontrol edilemeyen kardiyovasküler hastalıklar, 4. Son 6 ay içerisinde PF yönelik herhangi bir konservatif, girişimsel (enjeksiyonlar) ya da cerrahi tedavi almış olanlar, 5. Eksik takip. | |

**E. BAŞVURUDA YER ALAN KLİNİK ARAŞTIRMA MERKEZLERİ/ARAŞTIRMACILAR**

| **E.1** | **Koordinatör / sorumlu araştırmacı** *(çok / tek merkezli araştırmalar için)* | |
| --- | --- | --- |
| **E.1.1** | Adı Soyadı: | [Tuğba KOCAHAN ] |
| **E.1.2** | Ünvanı: | [ Doç.Dr. ] |
| **E.1.3** | Uzmanlık Alanı: | [Spor Hekimliği] |
| **E.1.4** | İş Adresi: | [SBÜ GEAH Spor Hekimliği Etlik Ankara] |
| **E.1.5** | E-Posta Adresi: | [ ] |
| **E.1.6** | GSM No: | [ ] |
| **E.1.7** | İmza: | [ ] |
|  | |  |

| **E.2** | **Yardımcı araştırmacı** *(Gerektiğinde bu bölümü tekrarlayınız.)* | |
| --- | --- | --- |
| **E.2.1** | Adı Soyadı: | [Aydan Örsçelik] |
| **E.2.2** | Ünvanı: | [ Doç. Dr. ] |
| **E.2.3** | Uzmanlık Alanı: | [ Spor Hekimliği ] |
| **E.2.4** | İş Adresi: | [ SBÜ GEAH Spor Hekimliği Etlik Ankara ] |
| **E.2.5** | E-posta Adresi: | [ ] |
| **E.2.6** | GSM No: | [ ] |
| **E.2.7** | **İmza:** | [ ] |

| **E.2** | **Yardımcı araştırmacı** *(Gerektiğinde bu bölümü tekrarlayınız.)* | |
| --- | --- | --- |
| **E.2.1** | Adı Soyadı: | [Bihter AKINOĞLU] |
| **E.2.2** | Ünvanı: | [ Doç.Dr. ] |
| **E.2.3** | Uzmanlık Alanı: | [Fizyoterapi] |
| **E.2.4** | İş Adresi: | [Yıldırım Beyazıt Üniversitesi Fizyoterapi Bölümü Ankara] |
| **E.2.5** | E-posta Adresi: | [ ] |
| **E.2.6** | GSM No: | [ ] |
| **E.2.7** | **İmza:** | [ ] |

| **E.3** | **Araştırmanın gerçekleştirileceği tesisler: Temel değerlendirme kriterlerinin ölçümü veya değerlendirilmesinin yapılacağı laboratuar veya diğer teknik tesisler** *(Birden çok kurum/ kuruluşsdolması halinde gerektiği gibi tekrarlayınız.)* | |
| --- | --- | --- |
| **E.3.1** | Kurum/Kuruluş: | [SBÜ GEAH Spor Hekimliği ] |
| **E.3.2** | Sorumlu Kişinin Adı Soyadı: | [Tuğba KOCAHAN] |
| **E.3.3** | Adresi: | [SBÜ GEAH Spor Hekimliği Etlik Ankara] |
| **E.3.4** | E-posta Adresi: | [ ] |
| **E.3.5** | GSM No: | [ ] |
| **E.3.6** | İmza: | [ ] |

**F. VERİ TOPLANMASI PLANLANAN YERLER** *(Gerektiğinde sayıyı arttırınız.)*

| **F. 1** | [SBÜ GEAH Spor Hekimliği Etlik Ankara ] |
| --- | --- |
| **F. 2** | [ ] |

**G. ARAŞTIRMA PLANI** *(İki sayfayı geçmeyecek şekilde doldurulmalıdır.)*

*(Araştırmanın insanlar üzerinde uygulanmasının gerekliliği, uygulamanın daha önce ülkemizde veya başka ülkelerde yapılıp yapılmadığı, yapılmışsa bu çalışmadan beklenen ek veriler veya bu çalışmanın diğerlerinden farkları, beklenen yararları, bilimsel veriler çerçevesinde açıklanmalıdır.)*

| **G.1** | **Amaç:** | [Bu araştırmanın ana amacı, Plantar fasiitis (PF) tedavisinde ekstracorporeal şok dalga tedavisi (ESWT) ve Kinezyo bantlama (KT) tedavisinin birlikte uygulanmasının ağrı üzerine akut etkisini incelemektir.] |
| --- | --- | --- |
| **G.2** | **Konu:** | [Plantar fasiitis (PF); yetişkinlerde topuk ağrısının yaygın bir nedenidir. Her yıl 1 milyondan fazla kişiyi etkiler ve bunların üçte ikisinin aile hekimine gittiği tahmin edilmektedir. PF, hem sedanterleri hem de sporcuları etkiler. Aşırı ayak pronasyonu (pes planus), yüksek kemer (pes cavus), bacak uzunluk eşitsizliği, obezite, aşırı koşu, uzun süreli ayakta durma/çok yürüme gerektiren meslekler (askeri personel), sedanter yaşam tarzı, aşil tendonunun ve intrensek ayak kaslarının gerginliği PF gelişimi için risk faktörleridir. Uzun süre ayakta durmaktan veya koşmaktan kaynaklanan biyomekanik aşırı kullanımın, kalkaneusta mikro yırtıklar oluşturmasından kaynaklandığı düşünülmektedir.  Hastalar sabah ilk adımlarında veya uzun süreli oturma sonrasında topuk ağrısı ile başvurabilirler. Fizik muayenede palpasyon ile medial plantar kalkaneal bölgede keskin ağrı, etkilenen ayağın hiperpronasyonu (düztabanlık), ağrı nedeniyle etkilenen ayağın ekin pozisyonunda anormal yürüyüşü, pasif ayak bileği/birinci parmak dorsifleksiyonu ile proksimal plantar fasiadaki rahatsızlık hissi ortaya çıkabilir.  Konservatif tedaviler, ağrıyı gidermeye yardımcı olur. Topuk ağrısı devam ederse fizik tedavi modaliteleri düşünülür. Ancak bunların hangisinin daha etkin olduğuna dair yeterli kanıt yoktur (1).  Ekstrakorporeal şok dalgası tedavisi (ESWT)’nin, hiperstimülasyon analjezisi, neovaskülarizasyon ve iyileşme sürecinin indüksiyonu gibi etki mekanizmaları nedeniyle PF tedavisinde etkili olduğu gösterilmiştir (2). ESWT ile tedavi edilen hastaların diğer tedavi yöntemleriyle tedavi edilen hastalardan daha yüksek iyileşme oranlarına, ağrı ölçeklerinde azalma, işe dönüş süresinde azalma ve daha az komplikasyona sahip olduğunu gösterilmiştir (3). Bu nedenle ESWT, PF tedavisinde güvenli ve etkili bir yöntem olarak önerilmektedir (4).  Kinezyo bantlama (KT) tedavisinin, PF’li hastalarda ağrı düzeylerini ve yaşam kalitesini iyileştirdiği gösterilmiştir (5). ESWT tedavisi, PF en etkili tedavi yöntemlerinden biri olarak bilinmesine rağmen KT tedavileri de ayak biyomekaniğini düzelterek analjezik etki sağlar (6).  Plantar fasiitis (PF) tedavisinde tek başına ekstracorporeal şok dalga tedavisi (ESWT) ve Kinezyo bantlama (KT) tedavisinin ağrı üzerine analjezik etki sağladığı bilinmesine rağmen her iki tedavi modalitesinin birlikte uygulanmasının etkinliğini inceleyen çalışma sayısı sınırlıdır.  **Araştırmanın Evreni/Örneklemi:** Hastanemiz Spor Hekimliği Anabilim Dalına başvuran, 18-65 yaş arası kadın ve erkek PF tanısı alan hastalar.  **Araştırmanın Yapılacağı Zaman Aralığı:** EPK ve Etik kurul onayı alındıktan sonra bir yıl.  **Araştırmanın Kısıtlılıkları:** Yeterli hasta sayısına ulaşılamaması  **Hipotezler:**   1. Plantar fasiitiste (PF) ekstrakorporeal şok dalga tedavisi (ESWT) uygulamasının ağrı üzerine akut etkisi vardır. 2. Kinezyo bantlama (KT) tedavisi ağrıyı gidermede etkili bir yöntemdir. 3. Plantar fasiitiste (PF) akut dönemde ekstrakorporeal şok dalga tedavisine (ESWT) Kinesio Bantlama uygulamasının eklenmesi ile günlük yaşam aktiviteleri artacaktır. 4. Plantar fasiitiste (PF) ESWT+Kinesio Bantlama uygulaması, tek başına ESWT uygulamasına göre kas kuvvetini arttıracaktır.   *Tüm bu bilgiler ışığında,* bu araştırmanın ana amacı, Plantar fasiitis (PF) tedavisinde ekstracorporeal şok dalga tedavisi (ESWT) ve Kinezyo bantlama (KT) tedavisinin birlikte uygulanmasının ağrı üzerine akut etkisini incelemektir]. |
| **G.3** | **Yöntem:** | Plantar fasiitis (PF) yetişkinlerde topuk ağrısının yaygın bir nedenidir. Her yıl 1 milyondan fazla kişiyi etkiler ve bunların üçte ikisinin aile hekimine gittiği tahmin edilmektedir. PF, hem sedanterleri hem de sporcuları etkiler. Konservatif tedaviler, ağrıyı gidermeye yardımcı olur. Özellikle ekstrakorporeal şok dalga tedavisi (ESWT) PF tedavisinde sıklıkla kullanılmakla birlikte Kinezyo bantlama (KT) tedavisinin de ağrıyı gidermede etkili bir yöntem olduğu bildirilmektedir. Literatürde her iki tedavi modalitesinin birlikte kullanıldığı çalışma sayısı sınırlıdır.  Hastanemiz Spor Hekimliği Anabilim Dalına PF hastaları sıklıkla başvurmaktadır. Bu nedenle bu hastaların tedavi edilmesi ve en uygun tedavinin belirlenmesi amacıyla hastanemize başvuran PF’li hastalara uygulama yaparak çalışma yapılmasına karar verilmiştir.  Bu çalışmada tedavi öncesi ve sonrası sonuçların karşılaştırılması ve gruplar arası farklılığın incelenmesi amacıyla yapılacak analiz için gerekli olan en küçük örneklem büyüklüğünü hesaplamak amacıyla G*Power 3.1.9.7 programı kullanılmıştır. Bağımsız iki örneklem (grup) karşılaştırması için G*Power programının bağımsız iki ortalama arası farklar (means: difference between two independent means) ile ilgili kısmı kullanılmıştır. Testin gücü %80, hata payı %5 ve etki büyüklüğü 0.80 (büyük) olarak alındığında toplam örneklem büyüklüğü 52 olarak hesaplandığı için çalışmaya her grupta en az 26 kişi olması ve toplam 52 kişi ile çalışmanın sonlandırılması planlanmaktadır.  Etik kurul onayı alındıktan sonraki üç ay içerisinde kliniğimize başvuran ve çalışma kriterlerine uygun olan hastalar çalışmaya dahil edilecektir. Çalışmaya dahil edilen PF hastalarına haftada bir gün toplam 4 seans olacak şekilde randomize olarak ESWT/ESWT+Kinesio Bantlama uygulaması yapılacaktır. Hastalar tedaviye ilk geldikleri gün ve son tedavi gününde ağrı değerlendirmesi, Ayak Fonksiyon Indeksi, Gastrosoleus ve plantar fasia esneklik ölçümü, Ayak bileği NEH, Fonksiyonel Testler/Heel Rise Testi, ve Pes Planus Ölçümü yapılacaktır.  İlk değerlendirmenin ardından hastalar spor hekimi tarafından yaş, boy, vücut ağırlığı ve vücut kitle indeksine göre alt gruplara ayrılacaktır. Benzer özelliklere sahip hastalar iki gruptan birine randomize edilecektir. |
| **G.4** | **Araştırma Akış Şeması** | [1. Etik kurul alınmasından itibaren GEAH Spor Hekimliği polikliniğine başvurup PF tanısı alan hastaların saptanması  2. Çalışmaya dahil edilecek hastaların tespiti ve gruplara ayrılması  3. Hastaların ilk geldikleri gün ağrı değerlendirmesi, Ayak Fonksiyon Indeksi, Gastrosoleus ve plantar fasia esneklik ölçümü, Ayak bileği NEH, Fonksiyonel Testler/Heel Rise Testi, ve Pes Planus Ölçümü  4. Hasataların tedavilerinin 4 hafta sürdürülmesi  5. Hastaların son tedavi günü ağrı değerlendirmesi, Ayak Fonksiyon Indeksi, Gastrosoleus ve plantar fasia esneklik ölçümü, Ayak bileği NEH, Fonksiyonel Testler/Heel Rise Testi, ve Pes Planus Ölçümü ] |
| **G.5** | **Kaynaklar:** | 1. Goff JD, Crawford R. Diagnosis and treatment of plantar fasciitis. Am Fam Physician. 2011; 84(6): 676-82. 2. [R L Roerdink](https://pubmed.ncbi.nlm.nih.gov/?term=Roerdink+RL&cauthor_id=28890412), [M Dietvorst](https://pubmed.ncbi.nlm.nih.gov/?term=Dietvorst+M&cauthor_id=28890412)  [B van der Zwaard](https://pubmed.ncbi.nlm.nih.gov/?term=van+der+Zwaard+B&cauthor_id=28890412), [H van der Worp](https://pubmed.ncbi.nlm.nih.gov/?term=van+der+Worp+H&cauthor_id=28890412), [J Zwerver](https://pubmed.ncbi.nlm.nih.gov/?term=Zwerver+J&cauthor_id=28890412). Complications of extracorporeal shockwave therapy in plantar fasciitis: Systematic review. Int J Surg. 2017 Oct;46:133-145. doi: 10.1016/j.ijsu.2017.08.587. 3. [Kai Sun](https://pubmed.ncbi.nlm.nih.gov/?term=Sun+K&cauthor_id=30502222), [Haiyu Zhou](https://pubmed.ncbi.nlm.nih.gov/?term=Zhou+H&cauthor_id=30502222), [Wenxue Jiang](https://pubmed.ncbi.nlm.nih.gov/?term=Jiang+W&cauthor_id=30502222). Extracorporeal shock wave therapy versus other therapeutic methods for chronic plantar fasciitis. Foot Ankle Surg. 2020 Jan;26(1):33-38. doi: 10.1016/j.fas.2018.11.002. 4. [Ching-Jen Wang](https://www.ncbi.nlm.nih.gov/pubmed/?term=Wang%20CJ%5BAuthor%5D&cauthor=true&cauthor_uid=22433113). Extracorporeal shockwave therapy in musculoskeletal disorders. [J Orthop Surg Res.](https://www.ncbi.nlm.nih.gov/pmc/articles/PMC3342893/) 2012; 7: 11. 5. [Nihal Tezel](https://pubmed.ncbi.nlm.nih.gov/?term=Tezel+N&cauthor_id=32952509), [Ebru Umay](https://pubmed.ncbi.nlm.nih.gov/?term=Umay+E&cauthor_id=32952509), [Musa Bulut](https://pubmed.ncbi.nlm.nih.gov/?term=Bulut+M&cauthor_id=32952509), [Aytul Cakci](https://pubmed.ncbi.nlm.nih.gov/?term=Cakci+A&cauthor_id=32952509). Short-Term Efficacy of Kinesiotaping versus Extracorporeal Shockwave Therapy for Plantar Fasciitis: A Randomized Study. Saudi J Med Med Sci. Sep-Dec 2020;8(3):181-187. doi: 10.4103/sjmms.sjmms_624_19. 6. [Yeliz Bahar-Ozdemir](https://pubmed.ncbi.nlm.nih.gov/?term=Bahar-Ozdemir+Y&cauthor_id=33410228), [Tugba Atan](https://pubmed.ncbi.nlm.nih.gov/?term=Atan+T&cauthor_id=33410228). Effects of adjuvant low-dye Kinesio taping, adjuvant sham taping, or extracorporeal shockwave therapy alone in plantar fasciitis: A randomised double-blind controlled trial. Int J Clin Pract. 2021 May;75(5):e13993. doi: 10.1111/ijcp.13993. |

**H. İLGİLİ BELGELER** *(Bu bölümde belirtilen belgeler sırası ile başvuru dosyasına eklenmelidir.)*

| **H.1** | **Eğitim Sorumlusu tarafından onaylanan belge***  ^*^Araştırmanın uzmanlık tezi ise, EPK tarafından onaylanan ıslak imzalı belge sunulmalıdır. |
| --- | --- |
| **H.2** | **Resmi Uygunluk Yazıları** |
| **H.3** | **Bilgilendirilmiş Gönüllü Olur Formu/Formları (BGOF)**  (Varsa, kontrol grubu olarak, sağlıklı gönüllüler için de BGOF örneği hazırlanmalıdır.  Pediatrik çalışmalarda, hem çocuklar (okuma-yazma bilen) ve hem de ebeveynleri için ayrı ayrı hazırlanan BGOF örnekleri, başvuru dosyasında yer almalıdır.  EK’te sunulmuştur. |
| **H.4** | **Biyolojik Materyal Transfer Formu (BMTF)** *(varsa)* |
| **H.5** | **Araştırma bütçesi** *(Araştırma giderlerinin tahmini bütçesini ve nasıl karşılanacağını belirtiniz.)*  *Araştırma 1000 tl civarnda kırtasiye gideri olacağı değerlendirilmektedir, bu da sorumlu araştırmacı tarafından karşılanacaktır.*  *Tedavi cihazları PF tedavisinde rutin olarak kullanılmaktadır. Hastalar mesai dışında tedaviye alınacaktır.* |
| **H.6** | **Özgeçmiş Formu** (sorumlu ve yardımcı araştırmacılar için)  **ÖZGEÇMİŞ FORMU***  **Tüm araştırmacılar için ayrı ayrı doldurulmalıdır*   1. **KİŞİSEL BİLGİLER**   A.1. Adı soyadı: Bihter AKINOĞLU  A.2. Unvanı: Doç.Dr.  A.3. Görev yeri: Ankara Yıldırım Beyazıt Üniversitesi  A.4 İletişim bilgileri *(e-posta adresi / telefon)*:   1. **EĞİTİM BİLGİLERİ**   B.1. Mezun olduğu üniversite / fakülte: Hacettepe Üniversitesi  B.2. Varsa uzmanlık Alanı:Fizyoterapi  B.3. Varsa, akademik ünvanları: Doç.Dr.   1. **İŞ TECRÜBESİNE AİT BİLGİLER**   C.1. Bugüne kadar çalıştığı kurum / kuruluşlar: Yıldırım Beyazıt Üniversitesi   1. **AKADEMİK ÇALIŞMALAR İLE İLGİLİ BİLGİLER**   D.1. Belirtmek istediğiniz önemli makaleleriniz *(en fazla beş makale)*:   1. Kocahan, T., Akınoğlu, B., Yilmaz, A. E., Rosemann, T., & Knechtle, B. (2021). Intra-and Inter-Rater Reliability of a Well-Used and a Less-Used IsoMed 2000 Dynamometer for Knee Flexion and Extension Peak Torque Measurements in a Concentric Test in Athletes. *Applied Sciences*, *11*(11), 4951. 2. Akınoğlu B, Kocahan T. Russian current versus high voltage current with isokinetic training on the quadriceps muscle strength and endurance. J Exerc Rehabil. 2020 Jun 30;16(3):272-278. doi: 10.12965/jer.2040260.130. PMID: 32724785; PMCID: PMC7365730. 3. Akınoğlu, B., Ünüvar, E., Kocahan, T., & Hasanoğlu, A. (2020). The Acute Effect of Nerve-Gliding Exercises on the Handgrip Strength of Adolescent Tennis Players. *Turkiye Klinikleri Spor Bilimleri*, *12*(3). 4. Akınoğlu B, Köse N. A comparison of the acute effects of radial extracorporeal shockwave therapy, ultrasound therapy, and exercise therapy in plantar fasciitis. J Exerc Rehabil. 2018 Apr 26;14(2):306-312. doi: 10.12965/jer.1836048.024. PMID: 29740568; PMCID: PMC5931170. 5. Akinoglu B, Köse N, Kirdi N, Yakut Y. Comparison of the Acute Effect of Radial Shock Wave Therapy and Ultrasound Therapy in the Treatment of Plantar Fasciitis: A Randomized Controlled Study. Pain Med. 2017 Dec 1;18(12):2443-2452. doi: 10.1093/pm/pnx113. PMID: 28575496.     D.2. Görev aldığınız projeler ve projedeki göreviniz *(en fazla beş proje):*  TARİH:  İMZA**:  **ÖZGEÇMİŞ FORMU***   1. **KİŞİSEL BİLGİLER**   A.1. Adı soyadı: Aydan ÖRSÇELİK  A.2. Unvanı:Doç. Dr.  A.3. Görev yeri: SBÜ GEAH Spor Hekimliği  A.4 İletişim bilgileri *(e-posta adresi / telefon)*:   1. **EĞİTİM BİLGİLERİ**   B.1. Mezun olduğu üniversite / fakülte: GATA  B.2. Varsa uzmanlık Alanı:Spor Hekimliği  B.3. Varsa, akademik ünvanları: Doç.Dr..   1. **İŞ TECRÜBESİNE AİT BİLGİLER**   C.1. Bugüne kadar çalıştığı kurum / kuruluşlar: TSK 2002- 2015, GEAH 2015-2017, SBÜ 2017-   1. **AKADEMİK ÇALIŞMALAR İLE İLGİLİ BİLGİLER**   D.1. Belirtmek istediğiniz önemli makaleleriniz *(en fazla beş makale)*:  1. Aydın, C. G., Örsçelik, A., Gök, M. C., & Akman, Y. E. (2020). The efficacy of extracorporeal shock wave therapy for chronic coccydynia. *Medical Principles and Practice*, *29*(5), 444-450.  2. APAYDIN, A. H., ÖRSÇELİK, A., & YILDIZ, Y. (2018). The effects of prolotherapy in recreational athletes with plantar fasciitis. *Spor Hekimliği Dergisi*, *53*(1), 37-46.  3. ÖRSÇELİK, A. (2015). Kas-İskelet Sistemi Yaralanmaları: Aşırı Kullanım Yara lanmaları. *Turkiye Klinikleri J Sports Med-Special Topics*, *1*(3), 62-9.  4. ÖRSÇELİK, A. (2016). Fonksiyonel Ayak Bileği İnstabilitesi Etyopatogenezi. Spor Hekimliği Dergisi, 51(3), 094-098.  TARİH:  İMZA**:  D.2. Görev aldığınız projeler ve projedeki göreviniz *(en fazla beş proje):*  **ÖZGEÇMİŞ FORMU***   1. **KİŞİSEL BİLGİLER**   A.1. Adı soyadı: Tuğba KOCAHAN  A.2. Unvanı: Doç.Dr.  A.3. Görev yeri: SBÜ GÜLHANE EĞİTİM ARAŞTIRMA HASTANESİ SPOR HEKİMLİĞİ AD  A.4 İletişim bilgileri *(e-posta adresi / telefon)*:   1. **EĞİTİM BİLGİLERİ**   B.1. Mezun olduğu üniversite / fakülte: Ankara Üniversitesi  B.2. Varsa uzmanlık Alanı:Spor Hekimliği  B.3. Varsa, akademik ünvanları: Doç.Dr.   1. **İŞ TECRÜBESİNE AİT BİLGİLER**   C.1. Bugüne kadar çalıştığı kurum / kuruluşlar: SESAM   1. **AKADEMİK ÇALIŞMALAR İLE İLGİLİ BİLGİLER**   D.1. Belirtmek istediğiniz önemli makaleleriniz *(en fazla beş makale)*:  1. Akınoğlu, B., & Kocahan, T. (2018). Comparison of muscular strength and balance in athletes with visual impairment and hearing impairment. *Journal of exercise rehabilitation*, *14*(5), 765.  2. Kocahan, T., & Akınoğlu, B. (2018). Determination of the relationship between core endurance and isokinetic muscle strength of elite athletes. *Journal of exercise rehabilitation*, *14*(3), 413.  3. Balcı, A., Akınoğlu, B., Kocahan, T., & Hasanoğlu, A. (2021). The relationships between isometric muscle strength and respiratory functions of the Turkish National Paralympic Goalball Team. *Journal of Exercise Rehabilitation*, *17*(1), 45.  4. Kocahan, T., Akınoğlu, B., Yilmaz, A. E., Rosemann, T., & Knechtle, B. (2021). Intra-and Inter-Rater Reliability of a Well-Used and a Less-Used IsoMed 2000 Dynamometer for Knee Flexion and Extension Peak Torque Measurements in a Concentric Test in Athletes. *Applied Sciences*, *11*(11), 4951.  D.2. Görev aldığınız projeler ve projedeki göreviniz *(en fazla beş proje):*  TARİH:  İMZA**:    ***Birden çok sayfa olması durumunda tüm sayfalar imzalanmalıdır* |
| **H.7** | **Ölçek ve/veya Anket Formu** *(varsa) Ek’te sunulmuştur* |
| **H.8** | **Hasta Takip Formu Örneği** *(varsa)*  Ekte sunulmuştur. |
| **H.9** | **Sigorta (Gerekiyorsa)** |

1. **TAAHHÜTNAME**

| **I.1** | **İşbu başvuru formuyla, başvuru sahibi (koordinatör/sorumlu araştırmacı) olarak;** |
| --- | --- |
|  | - Bu araştırmanın eş zamanlı olarak başka bir etik kurula sunulmadığını, - Başvuru dosyasında yer alan bilgilerin doğru olduğunu, - Araştırmanın protokole, ilgili mevzuata, güncel kılavuzlara, güncel Helsinki Bildirgesi ve İyi Klinik Uygulamaları ilkelerine uygun olarak gerçekleştirileceğini, - Önerilen klinik araştırmanın gerçekleştirilebilir nitelikte olduğunu, - Araştırma ekibini (laborutuvar ekibi dahil) araştırma hakkında bilgilendirdiğimi, - Araştırmanın Etik Kurul tarafından onaylandığı tarihten itibaren 6 ( altı ) ay içinde başlatılmadığı veya araştırmadan vazgeçildiği durumda, konu ile ilgili olarak Kurulunuzu bilgilendireceğimi, - Araştırma ekibinde ortaya çıkacak değişiklikler ve araştırma protokolünde amaç, yöntem vb gibi değişikliklerde, sözkonusu değişikliğin gerçekleştirilmesinden önce, durumu Kurulunuza bildireceğimi, - Araştırmanın her türlü maddi (destekleyicinin karşıladığının dışındaki giderler) ve hukuki sorumluluğunu üstlendiğimi, taahhüt ederim. |
| **I.2** | **Başvuru sahibi** **(koordinatör/sorumlu araştırmacı)** (Bu bölüm, el yazısı ile doldurulacaktır.)  (Tez projelerinde başvuru sahibi, danışman öğretim üyesi olmalıdır.) |
| **I.2.1** | **Adı Soyadı:** [ ] |
| **I.2.2** | **Tarih (gün/ay/yıl):** [ ] |
| **I.2.3** | **İmza:** [ ] |
