# Supplementary material for: Can kinesio tape negatively affect the treatment by creating a hard floor in plantar fasciitis treatment? A randomized clinical trial
Source: PLoS One. 2025 May 5;20(5):e0322397. doi: 10.1371/journal.pone.0322397 (PMC12052111; doi:10.1371/journal.pone.0322397)
Supplement: S2 Study Protocol — (DOCX) [file pone.0322397.s003.docx]

**CLINICAL RESEARCH ETHICS COMMITTEE**

*(Kırmızı çerçeve içindeki alanlar Klinik Araştırmalar Etik Kurulu Sekretaryası tarafından doldurulacaktır*).

(Fields in the red frame will be filled in by the Clinical Research Ethics Committee Secretariat).

| Başvurunun yapıldığı tarih: [ ] |  | Red/olumsuz görüş nedenleri: [ ]  Tarih: [ ] |  |
| --- | --- | --- | --- |
| Başvuru arşiv kayıt numarası: | | |  |
| Düzeltme yapıldı ise tarihi: | | |  |

**ETHICS COMMITTEE INFORMATION**

| **1.** | **Has an Ethics Committee application been made before for research approval? NO** |  |  |
| --- | --- | --- | --- |
| **1.1.** | If your answer is yes: | | |
| **1.1.1.** | Name of the Ethics Committee: [ ] | | |
| **1.1.2.** | Application date: [ ] | | |

**A. RESEARCH**

| **A.1** | **Name of all researchers**: [Doç.Dr. Tuğba KOCAHAN, Doç.Dr. Aydan ÖRSÇELİK, Doç.Dr. Bihter AKINOĞLU] | | | |
| --- | --- | --- | --- | --- |
| **A.2** | **Name of the research (Abbreviations should not be used and should reflect the purpose of the research):** [Examining the Effectiveness of Kinesiotaping Applications Applied with Extracorporeal Shock Wave Therapy (ESWT) in Plantar Fasciitis] | | | |
| **A.3** | **Is the study part of the pediatric research plan? NO** | **** | **** | |
| **A.4** | **Status of the research (Check the appropriate box below.)** | | | |
| **A.4.1** | Master's Thesis | | |  |
| **A.4.2** | PhD Thesis | | |  |
| **A.4.3** | Medical Specialization Thesis | | |  |
| **A.4.4** | Individual Research Project | | |  |
| **A.4.5** | Multicenter research | | |  |
| **A.4.6** | Other (specify): [ ] | | | |

**B. SUPPORTIVE**

| **B.1.** | **Is there a sponsor for the research** (current/planned)**? NO** |  |  | |
| --- | --- | --- | --- | --- |
| **B.1.1** | **B.1**’e cevabınız evet ise, aşağıdaki uygun kutucuğu işaretleyiniz. | | | |
| **B.1.1.1** | Üniversite (Bilimsel Araştırma Projeleri Koordinasyon Birimi; *BAP*) | | |  |
| **B.1.1.2** | Eğitim ve Araştırma Hastanesi | | |  |
| **B.1.1.3** | TÜBİTAK *(Türkiye Bilimsel ve Teknolojik Araştırma Kurumu)* | | |  |
| **B.1.1.4** | DPT *(Devlet Planlama Teşkilatı)* | | |  |
| **B.1.1.5** | Uluslararası ise belirtiniz: [ ] | | | |
| **B.1.1.6** | Diğer ise (özel kuruluş, vb), belirtiniz: [ ] | | | |

**C. GENERAL INFORMATION ABOUT THE RESEARCH**

| **C.1** | | **Medical condition or disease under investigation** | |
| --- | --- | --- | --- |
| **C.1.1** | | Specify the medical condition or disease investigated (state in free text):  [Patients diagnosed with plantar fasciitis] | |
| **C.1.1.1** | | Specify field of study (physiology, field work, oncology, hematology, etc.):  [ Sports Medicine, Physiotherapy] | |
| **C.2** | **Type of research** (Check the appropriate box/boxes.) | | |
| **C.2.1** | Retrospective studies based on archival scans, such as files and image recordings |  | |
| **C.2.2** | Research conducted with health-related surveys and similar data collection tools |  | |
| **C.2.3** | Research in which data collected through computer-based tests, interviews, and audio/video recordings will be used. |  | |
| **C.2.4** | **Non-drug observational studies:**Tanımlayıcı  Kesitsel  Olgu-Kontrol  Kohort |  | |
| **C.2.5** | Research conducted with biochemistry, microbiology, pathology and radiology materials such as blood, urine, tissue, hair, feathers, saliva, stool and radiological images. |  | |
| **C.2.6** | Studies conducted with materials obtained during routine examination, analysis and treatment procedures |  | |
| **C.2.7** | Research on body physiology, such as exercise |  | |
| **C.2.8** | Research to be conducted within the boundaries of nursing activities |  | |
| **C.2.9** | Cell or tissue culture (in vitro) research |  | |
| **C.2.10** | Studies to be carried out with genetic material other than gene therapy and aimed at identification |  | |
| **C.2.11** | Research based on anthropometric measurements |  | |
| **C.2.12** | Studies to evaluate life habits such as nutrition |  | |
| **C.2.13** | If other, specify: [Examining the effectiveness of applying two different treatments together] |  | |

| **C.3** | **Design of the research** | | |
| --- | --- | --- | --- |
| **C.3.1** | Controlled (research with a control group) **YES** |  |  |
| **C.3.2** | If other, specify: [ ] | | |

| **C.4** | **Research center** | | | |
| --- | --- | --- | --- | --- |
| **C.4.1** | There is only one center **YES** |  |  | |
| **C.4.2** | There are multiple centers **NO** |  |  | |
| **C.4.2.1** | Please indicate the number and names of the centers envisaged in our country: [SBU GEAH Sports Medicine] | | | |
| **C.4.3** | Will this research be conducted in other countries? **NO** |  |  | |
| **C.4.3.1** | **If your answer to C.4.3 is yes, please specify the number of centers and countries envisaged in other countries:** | | | |
| **C.4.4** | Is it planned to send the biological materials collected within the scope of the study to another institution in the country or abroad? **NO** |  |  | |
| **C.4.4.1** | **If your answer to C.4.4 is yes, explain: [ ]**  Attach the current biological material transfer agreement at address [www.titck.gov.tr](http://www.titck.gov.tr) to the application file. | | | |
| **C.5** | **Research duration** (in days, months and years): | | | |
| **C.5.1.** | Please indicate how long the research will take in your country.: | [ ] | [ ] | [One year] |
| **C.5.1.2** | If so, indicate the estimated length of time the research will take in all countries included in the research: | [ ] | [ ] | [ ] |
| **C.6** | **Recommended date to start recruiting volunteers for the study** (in day, month and year): | | | |
| **C.6.1** | Please indicate the history in our country: | [01 ] | [08] | [2023] |
| **C.6.1.2** | If available, please specify date in other countries: | [ ] | [ ] | [ ] |

| **C.7** | **Data collection method used in the research** (Check the appropriate box/boxes.) | |
| --- | --- | --- |
| **C.7.1** | Questionnaire |  |
| **C.7.2** | Interview |  |
| **C.7.3** | Observation |  |
| **C.7.4** | File scan (retrospective) |  |
| **C.7.5** | Test application in computer environment |  |
| **C.7.6** | image recording |  |
| **C.7.7** | voice recording |  |
| **C.7.7.1** | Study on biological material | |
| **C.7.7.2** | In vivo study |  |
| **C.7.7.3** | In vitro study |  |
| **C.7. 8** | Ex vivo study |  |
| **C.7.9** | Post-mortem |  |
| **C.7.10** | If other, please specify…: [ ] | |

**D. VOLUNTEERS TAKEN INTO THE RESEARCH**

| **D.1** | **Age range** (Indicate the estimated number of volunteers planned in each age range for the entire research.) | | |
| --- | --- | --- | --- |
| **D.1.1** | Under 18 years old |  |  |
| **D.1.1.1** | If your answer to **D.1.1** is yes, please specify the age range and number of volunteers: [] | | |
| **D.1.2** | Over 18: [ 44 ] **YES** |  |  |
| **D.1.2.1** | If your answer to **D.1.2** is yes, please specify the age range and number of volunteers: All age groups that applied | | |

| **D.2** | **Gender** | |
| --- | --- | --- |
| **D.2.1** | Woman |  |
| **D.2.2** | Male |  |

| **D.3** | **Volunteer group in the study** | |
| --- | --- | --- |
| **D.3.1** | Healthy volunteers |  |
| **D.3.2** | Patients |  |
| **D.3.3** | Special sensitive groups |  |
| **D.3.3.1** | Women of childbearing potential who do not use contraception |  |
| **D.3.3.2** | Women who use birth control and are of childbearing potential |  |
| **D.3.3.3** | Pregnant women |  |
| **D.3.3.4** | Breastfeeding women |  |
| **D.3.3.5** | Urgent cases |  |
| **D.3.3.6** | Volunteers who cannot give consent in person |  |
| **D.3.3.6.1** | If your answer to **D.3.3.6 is yes, please specify: [ ]** | |
| **D.3.3.7** | If other, specify: [ ] | |
| **D.4** | **Criteria for including volunteers in the study (List in items).**  (If available, separate rankings should be made for healthy volunteers as a control group). | |
|  | 1. Being diagnosed with PF  2. Not having any known systemic problems,  3. Not having undergone any surgical operation on the lower extremity,  4. Not having had a lower extremity injury in the last three months,  5. Being between the ages of 18-65  6. Men and women volunteering. | |
| **D.5** | **Criteria for not including volunteers in the study (List them in items.)**  (If available, separate sequencing should be done for healthy volunteers as a control group.) | |
|  | 1. Pregnancy,  2. Bleeding disorder,  3. Uncontrolled cardiovascular diseases,  4. Those who have received any conservative, interventional (injections) or surgical treatment for PF in the last 6 months,  5. Incomplete follow up. | |

**E. CLINICAL RESEARCH CENTERS/RESEARCHERS INCLUDED IN THE APPLICATION**

| **E.1** | **Coordinator/principal investigator** (for multi/single center studies) | |
| --- | --- | --- |
| **E.1.1** | Name and surname: | [Tuğba KOCAHAN ] |
| **E.1.2** | Title: | [Assoc. Dr.] |
| **E.1.3** | Profession: | [Sports Medicine] |
| **E.1.4** | Business Address: | [SBU GEAH Sports Medicine Etlik Ankara] |
| **E.1.5** | E-mail address: | [ ] |
| **E.1.6** | Phone number | [ ] |
| **E.1.7** | Signature: | [ ] |
|  | |  |

| **E.2** | **Assistant researcher** (Repeat this section when necessary.) | |
| --- | --- | --- |
| **E.2.1** | Title: | [Aydan Örsçelik] |
| **E.2.2** | Profession: | [Assoc. Dr.] |
| **E.2.3** | Business Address: | [Sports Medicine] |
| **E.2.4** | E-mail address: | [SBU GEAH Sports Medicine Etlik Ankara ] |
| **E.2.5** | Phone number | [ ] |
| **E.2.6** | Signature: | [ ] |
| **E.2.7** | Title: | [ ] |

| **E.2** | **Assistant researcher** (Repeat this section when necessary.) | |
| --- | --- | --- |
| **E.2.1** | Title: | [Bihter AKINOĞLU] |
| **E.2.2** | Profession: | [Assoc. Dr. |
| **E.2.3** | Business Address: | [Physiotherapy] |
| **E.2.4** | E-mail address: | [Yıldırım Beyazıt University Physiotherapy Department Ankara] |
| **E.2.5** | Phone number | [ ] |
| **E.2.6** | Signature: | [ ] |
| **E.2.7** | Title: | [ ] |

| **E.3** | **Facilities where the research will be carried out: Laboratory or other technical facilities where the measurement or evaluation of the basic evaluation criteria will be carried out** (Repeat as necessary if more than one institution/organization is involved.) | |
| --- | --- | --- |
| **E.3.1** | Institution / University: | [SBU GEAH Sports Medicine] |
| **E.3.2** | Name and Surname of the Responsible Person: | [Tuğba KOCAHAN] |
| **E.3.3** | Address: | [SBU GEAH Sports Medicine Etlik Ankara] |
| **E.3.4** | E mail address: | [ ] |
| **E.3.5** | Phone number: | [ ] |
| **E.3.6** | Signature: | [ ] |

**F. PLACES PLANNED TO COLLECT DATA** *(Gerektiğinde sayıyı arttırınız.)*

| **F. 1** | [SBÜ GEAH Sports Medicine Etlik Ankara] |
| --- | --- |
| **F. 2** | [ ] |

**G. RESEARCH PLAN (Must be filled in no more than two pages.)**

*(Araştırmanın insanlar üzerinde uygulanmasının gerekliliği, uygulamanın daha önce ülkemizde veya başka ülkelerde yapılıp yapılmadığı, yapılmışsa bu çalışmadan beklenen ek veriler veya bu çalışmanın diğerlerinden farkları, beklenen yararları, bilimsel veriler çerçevesinde açıklanmalıdır.)*

| **G.1** | **Aim:** | The main purpose of this study is to examine the acute effect of the combined application of extracorporeal shock wave therapy (ESWT) and Kinesio taping (KT) therapy on pain in the treatment of Plantar fasciitis (PF). |
| --- | --- | --- |
| **G.2** | **Subject:** | Plantar fasciitis (PF); It is a common cause of heel pain in adults. It affects more than 1 million people each year, and it is estimated that two-thirds of them see their family doctor. PF affects both sedentary people and athletes. Excessive foot pronation (pes planus), high arch (pes cavus), leg length inequality, obesity, excessive running, professions that require long periods of standing/a lot of walking (military personnel), sedentary lifestyle, tension of the Achilles tendon and intrinsic foot muscles are risk factors for the development of PF. It is thought to be caused by biomechanical overuse from standing or running for long periods of time, creating micro-tears in the calcaneus.  Patients may present with heel pain during their first steps in the morning or after sitting for a long time. Physical examination may reveal sharp pain in the medial plantar calcaneal region upon palpation, hyperpronation of the affected foot (flat feet), abnormal gait of the affected foot in the equinus position due to pain, passive ankle/first toe dorsiflexion, and discomfort in the proximal plantar fascia.  Conservative treatments help relieve pain. If heel pain persists, physical therapy modalities are considered. However, there is not enough evidence as to which of these is more effective (1).  ESWT has been shown to be effective in the treatment of PF due to its mechanisms of action such as hyperstimulation analgesia, neovascularization and induction of the healing process (2). It has been shown that patients treated with ESWT have higher recovery rates, decreased pain scales, decreased time to return to work, and fewer complications than patients treated with other treatment methods (3).Therefore, ESWT is recommended as a safe and effective method in the treatment of PF (4).  Kinesio taping (KT) treatment has been shown to improve pain levels and quality of life in patients with PF (5). Although ESWT treatment is known as one of the most effective treatment methods for PF, KT treatments also provide an analgesic effect by improving foot biomechanics (6).  Although it is known that ESWT and KT therapy alone provide an analgesic effect on pain in the treatment of PF, the number of studies examining the effectiveness of both treatment modalities together is limited.  **Population/Sample of the Research:** Male and female patients diagnosed with PF between the ages of 18-65 who applied to our hospital's Sports Medicine Department.  **Time period in which the research will be carried out:** One year after EPK and Ethics committee approval is received.  **Limitations of the Study:** Inability to reach sufficient number of patients  **Hypotheses:**  **1**. Extracorporeal shock wave therapy (ESWT) application in plantar fasciitis (PF) has an acute effect on pain.  2. Kinesio taping (KT) treatment is an effective method for relieving pain.  3. Daily living activities will increase with the addition of Kinesio Taping to extracorporeal shock wave therapy (ESWT) in the acute phase of plantar fasciitis (PF).  4. ESWT+Kinesio Taping application in plantar fasciitis (PF) will increase muscle strength compared to ESWT application alone.  *In the light of all this information,* the main purpose of this study is to examine the acute effect of the combined application of ESWT and KT therapy on pain in the treatment of PF. |
| **G.3** | **Method:** | PF is a common cause of heel pain in adults. It affects more than 1 million people each year, and it is estimated that two-thirds of them see their family doctor. PF affects both sedentary people and athletes. Conservative treatments help relieve pain. Especially ESWT is frequently used in the treatment of PF, and KT treatment is also reported to be an effective method in relieving pain. The number of studies in the literature where both treatment modalities are used together is limited.  In this study, G*Power 3.1.9.7 program was used to calculate the smallest sample size required for analysis to compare the results before and after treatment and to examine the differences between groups. For the comparison of two independent samples (groups), the part of the G*Power program related to the differences between two independent means was used. Considering the power of the test as 80%, the margin of error as 5% and the effect size as 0.80 (large), the total sample size is calculated as 52. Therefore, it is planned to include at least 26 people in each group and to end the study with a total of 52 people.  PF patients frequently apply to our hospital's Department of Sports Medicine. For this reason, it was decided to conduct a study on patients with PF who applied to our hospital in order to treat these patients and determine the most appropriate treatment. Patients who apply to our clinic within three months after receiving ethics committee approval and meet the study criteria will be included in the study. ESWT/ESWT+Kinesio Taping will be applied randomly to PF patients included in the study, once a week for a total of 4 sessions. Pain evaluation, Foot Function Index, Gastrosoleus and plantar fascia flexibility measurement, Ankle ROM, Functional Tests/Heel Rise Test, and Pes Planus Measurement will be performed on the first day of treatment and on the last day of treatment.  After the initial evaluation, patients will be divided into subgroups by the sports physician according to age, height, body weight and body mass index. Patients with similar characteristics will be randomized into one of two groups. |
| **G.4** | **Research Flow Chart** | 1. After obtaining EPK and Ethics committee approval, identifying patients diagnosed with PF who applied to GEAH Sports Medicine outpatient clinic (2 months)  2. Identifying the patients to be included in the study and dividing them into groups (2 months)  3. On the first day of arrival, patients will be evaluated for pain, muscle strength measurement, and their daily living activities and function will be evaluated (2 months).  4. Continuing the treatment of the patients for 4 weeks (3 months)  5. At the end of treatment, patients will be evaluated for pain, muscle strength measurement, and daily living activities and function evaluation (2 months). |
| **G.5** | **References:** | 1. Goff JD, Crawford R. Diagnosis and treatment of plantar fasciitis. Am Fam Physician. 2011; 84(6): 676-82. 2. [R L Roerdink](https://pubmed.ncbi.nlm.nih.gov/?term=Roerdink+RL&cauthor_id=28890412), [M Dietvorst](https://pubmed.ncbi.nlm.nih.gov/?term=Dietvorst+M&cauthor_id=28890412)  [B van der Zwaard](https://pubmed.ncbi.nlm.nih.gov/?term=van+der+Zwaard+B&cauthor_id=28890412), [H van der Worp](https://pubmed.ncbi.nlm.nih.gov/?term=van+der+Worp+H&cauthor_id=28890412), [J Zwerver](https://pubmed.ncbi.nlm.nih.gov/?term=Zwerver+J&cauthor_id=28890412). Complications of extracorporeal shockwave therapy in plantar fasciitis: Systematic review. Int J Surg. 2017 Oct;46:133-145. doi: 10.1016/j.ijsu.2017.08.587. 3. [Kai Sun](https://pubmed.ncbi.nlm.nih.gov/?term=Sun+K&cauthor_id=30502222), [Haiyu Zhou](https://pubmed.ncbi.nlm.nih.gov/?term=Zhou+H&cauthor_id=30502222), [Wenxue Jiang](https://pubmed.ncbi.nlm.nih.gov/?term=Jiang+W&cauthor_id=30502222). Extracorporeal shock wave therapy versus other therapeutic methods for chronic plantar fasciitis. Foot Ankle Surg. 2020 Jan;26(1):33-38. doi: 10.1016/j.fas.2018.11.002. 4. [Ching-Jen Wang](https://www.ncbi.nlm.nih.gov/pubmed/?term=Wang%20CJ%5BAuthor%5D&cauthor=true&cauthor_uid=22433113). Extracorporeal shockwave therapy in musculoskeletal disorders. [J Orthop Surg Res.](https://www.ncbi.nlm.nih.gov/pmc/articles/PMC3342893/) 2012; 7: 11. 5. [Nihal Tezel](https://pubmed.ncbi.nlm.nih.gov/?term=Tezel+N&cauthor_id=32952509), [Ebru Umay](https://pubmed.ncbi.nlm.nih.gov/?term=Umay+E&cauthor_id=32952509), [Musa Bulut](https://pubmed.ncbi.nlm.nih.gov/?term=Bulut+M&cauthor_id=32952509), [Aytul Cakci](https://pubmed.ncbi.nlm.nih.gov/?term=Cakci+A&cauthor_id=32952509). Short-Term Efficacy of Kinesiotaping versus Extracorporeal Shockwave Therapy for Plantar Fasciitis: A Randomized Study. Saudi J Med Med Sci. Sep-Dec 2020;8(3):181-187. doi: 10.4103/sjmms.sjmms_624_19. 6. [Yeliz Bahar-Ozdemir](https://pubmed.ncbi.nlm.nih.gov/?term=Bahar-Ozdemir+Y&cauthor_id=33410228), [Tugba Atan](https://pubmed.ncbi.nlm.nih.gov/?term=Atan+T&cauthor_id=33410228). Effects of adjuvant low-dye Kinesio taping, adjuvant sham taping, or extracorporeal shockwave therapy alone in plantar fasciitis: A randomised double-blind controlled trial. Int J Clin Pract. 2021 May;75(5):e13993. doi: 10.1111/ijcp.13993. |

**H. RELATED DOCUMENTS** (The documents specified in this section must be added to the application file respectively.)

| **H.1** | **Document approved by the Education Officer***  *If the research is a specialization thesis, a signed document approved by EPK must be submitted. |
| --- | --- |
| **H.2** | **Official Letters of Conformity** |
| **H.3** | **Informed Volunteer Form(s) (BGOF)**  (If available, a BGOF sample should also be prepared for healthy volunteers as a control group.  In pediatric studies, BGOF samples prepared separately for both children (who can read and write) and their parents should be included in the application file.  **It is presented in the Appendix.** |
| **H.4** | **Biological Material Transfer Form (BMTF)** (if applicable) |
| **H.5** | **Research budget** (Indicate the estimated budget of research expenses and how they will be covered.)  It is estimated that the research will have a stationery cost of around 1000 TL, which will be covered by the responsible researcher.  Therapy devices are routinely used in the treatment of PF.  Patients will be treated outside of working hours. |
| **H.6** | **Resume Form** (for principal and assistant researchers)  **RESUME FORM***  *****Must be filled out separately for all researchers  **A. PERSONAL INFORMATION**  A.1. Adı soyadı: Bihter AKINOĞLU  A.2. Unvanı: Doç.Dr.  A.3. Görev yeri: Ankara Yıldırım Beyazıt Üniversitesi  A.4 İletişim bilgileri *(e-posta adresi / telefon)*:   1. **EĞİTİM BİLGİLERİ**   B.1. Mezun olduğu üniversite / fakülte: Hacettepe Üniversitesi  B.2. Varsa uzmanlık Alanı:Fizyoterapi  B.3. Varsa, akademik ünvanları: Doç.Dr.   1. **İŞ TECRÜBESİNE AİT BİLGİLER**   C.1. Bugüne kadar çalıştığı kurum / kuruluşlar: Yıldırım Beyazıt Üniversitesi   1. **AKADEMİK ÇALIŞMALAR İLE İLGİLİ BİLGİLER**   D.1. Belirtmek istediğiniz önemli makaleleriniz *(en fazla beş makale)*:   1. Kocahan, T., Akınoğlu, B., Yilmaz, A. E., Rosemann, T., & Knechtle, B. (2021). Intra-and Inter-Rater Reliability of a Well-Used and a Less-Used IsoMed 2000 Dynamometer for Knee Flexion and Extension Peak Torque Measurements in a Concentric Test in Athletes. *Applied Sciences*, *11*(11), 4951. 2. Akınoğlu B, Kocahan T. Russian current versus high voltage current with isokinetic training on the quadriceps muscle strength and endurance. J Exerc Rehabil. 2020 Jun 30;16(3):272-278. doi: 10.12965/jer.2040260.130. PMID: 32724785; PMCID: PMC7365730. 3. Akınoğlu, B., Ünüvar, E., Kocahan, T., & Hasanoğlu, A. (2020). The Acute Effect of Nerve-Gliding Exercises on the Handgrip Strength of Adolescent Tennis Players. *Turkiye Klinikleri Spor Bilimleri*, *12*(3). 4. Akınoğlu B, Köse N. A comparison of the acute effects of radial extracorporeal shockwave therapy, ultrasound therapy, and exercise therapy in plantar fasciitis. J Exerc Rehabil. 2018 Apr 26;14(2):306-312. doi: 10.12965/jer.1836048.024. PMID: 29740568; PMCID: PMC5931170. 5. Akinoglu B, Köse N, Kirdi N, Yakut Y. Comparison of the Acute Effect of Radial Shock Wave Therapy and Ultrasound Therapy in the Treatment of Plantar Fasciitis: A Randomized Controlled Study. Pain Med. 2017 Dec 1;18(12):2443-2452. doi: 10.1093/pm/pnx113. PMID: 28575496.     D.2. Görev aldığınız projeler ve projedeki göreviniz *(en fazla beş proje):*  TARİH:  İMZA**:  **ÖZGEÇMİŞ FORMU***   1. **KİŞİSEL BİLGİLER**   A.1. Adı soyadı: Aydan ÖRSÇELİK  A.2. Unvanı:Doç. Dr.  A.3. Görev yeri: SBÜ GEAH Spor Hekimliği  A.4 İletişim bilgileri *(e-posta adresi / telefon)*:   1. **EĞİTİM BİLGİLERİ**   B.1. Mezun olduğu üniversite / fakülte: GATA  B.2. Varsa uzmanlık Alanı:Spor Hekimliği  B.3. Varsa, akademik ünvanları: Doç.Dr..   1. **İŞ TECRÜBESİNE AİT BİLGİLER**   C.1. Bugüne kadar çalıştığı kurum / kuruluşlar: TSK 2002- 2015, GEAH 2015-2017, SBÜ 2017-   1. **AKADEMİK ÇALIŞMALAR İLE İLGİLİ BİLGİLER**   D.1. Belirtmek istediğiniz önemli makaleleriniz *(en fazla beş makale)*:  1. Aydın, C. G., Örsçelik, A., Gök, M. C., & Akman, Y. E. (2020). The efficacy of extracorporeal shock wave therapy for chronic coccydynia. *Medical Principles and Practice*, *29*(5), 444-450.  2. APAYDIN, A. H., ÖRSÇELİK, A., & YILDIZ, Y. (2018). The effects of prolotherapy in recreational athletes with plantar fasciitis. *Spor Hekimliği Dergisi*, *53*(1), 37-46.  3. ÖRSÇELİK, A. (2015). Kas-İskelet Sistemi Yaralanmaları: Aşırı Kullanım Yara lanmaları. *Turkiye Klinikleri J Sports Med-Special Topics*, *1*(3), 62-9.  4. ÖRSÇELİK, A. (2016). Fonksiyonel Ayak Bileği İnstabilitesi Etyopatogenezi. Spor Hekimliği Dergisi, 51(3), 094-098.  TARİH:  İMZA**:  D.2. Görev aldığınız projeler ve projedeki göreviniz *(en fazla beş proje):*  **ÖZGEÇMİŞ FORMU***   1. **KİŞİSEL BİLGİLER**   A.1. Adı soyadı: Tuğba KOCAHAN  A.2. Unvanı: Doç.Dr.  A.3. Görev yeri: SBÜ GÜLHANE EĞİTİM ARAŞTIRMA HASTANESİ SPOR HEKİMLİĞİ AD  A.4 İletişim bilgileri *(e-posta adresi / telefon)*:   1. **EĞİTİM BİLGİLERİ**   B.1. Mezun olduğu üniversite / fakülte: Ankara Üniversitesi  B.2. Varsa uzmanlık Alanı:Spor Hekimliği  B.3. Varsa, akademik ünvanları: Doç.Dr.   1. **İŞ TECRÜBESİNE AİT BİLGİLER**   C.1. Bugüne kadar çalıştığı kurum / kuruluşlar: SESAM   1. **AKADEMİK ÇALIŞMALAR İLE İLGİLİ BİLGİLER**   D.1. Belirtmek istediğiniz önemli makaleleriniz *(en fazla beş makale)*:  1. Akınoğlu, B., & Kocahan, T. (2018). Comparison of muscular strength and balance in athletes with visual impairment and hearing impairment. *Journal of exercise rehabilitation*, *14*(5), 765.  2. Kocahan, T., & Akınoğlu, B. (2018). Determination of the relationship between core endurance and isokinetic muscle strength of elite athletes. *Journal of exercise rehabilitation*, *14*(3), 413.  3. Balcı, A., Akınoğlu, B., Kocahan, T., & Hasanoğlu, A. (2021). The relationships between isometric muscle strength and respiratory functions of the Turkish National Paralympic Goalball Team. *Journal of Exercise Rehabilitation*, *17*(1), 45.  4. Kocahan, T., Akınoğlu, B., Yilmaz, A. E., Rosemann, T., & Knechtle, B. (2021). Intra-and Inter-Rater Reliability of a Well-Used and a Less-Used IsoMed 2000 Dynamometer for Knee Flexion and Extension Peak Torque Measurements in a Concentric Test in Athletes. *Applied Sciences*, *11*(11), 4951.  D.2. Görev aldığınız projeler ve projedeki göreviniz *(en fazla beş proje):*  TARİH:  İMZA**:    ***Birden çok sayfa olması durumunda tüm sayfalar imzalanmalıdır* |
| **H.7** | **Ölçek ve/veya Anket Formu** *(varsa) Ek’te sunulmuştur* |
| **H.8** | **Hasta Takip Formu Örneği** *(varsa)*  Ekte sunulmuştur. |
| **H.9** | **Sigorta (Gerekiyorsa)** |

1. **TAAHHÜTNAME**

| **I.1** | **İşbu başvuru formuyla, başvuru sahibi (koordinatör/sorumlu araştırmacı) olarak;** |
| --- | --- |
|  | - Bu araştırmanın eş zamanlı olarak başka bir etik kurula sunulmadığını, - Başvuru dosyasında yer alan bilgilerin doğru olduğunu, - Araştırmanın protokole, ilgili mevzuata, güncel kılavuzlara, güncel Helsinki Bildirgesi ve İyi Klinik Uygulamaları ilkelerine uygun olarak gerçekleştirileceğini, - Önerilen klinik araştırmanın gerçekleştirilebilir nitelikte olduğunu, - Araştırma ekibini (laborutuvar ekibi dahil) araştırma hakkında bilgilendirdiğimi, - Araştırmanın Etik Kurul tarafından onaylandığı tarihten itibaren 6 ( altı ) ay içinde başlatılmadığı veya araştırmadan vazgeçildiği durumda, konu ile ilgili olarak Kurulunuzu bilgilendireceğimi, - Araştırma ekibinde ortaya çıkacak değişiklikler ve araştırma protokolünde amaç, yöntem vb gibi değişikliklerde, sözkonusu değişikliğin gerçekleştirilmesinden önce, durumu Kurulunuza bildireceğimi, - Araştırmanın her türlü maddi (destekleyicinin karşıladığının dışındaki giderler) ve hukuki sorumluluğunu üstlendiğimi, taahhüt ederim. |
| **I.2** | **Başvuru sahibi** **(koordinatör/sorumlu araştırmacı)** (Bu bölüm, el yazısı ile doldurulacaktır.)  (Tez projelerinde başvuru sahibi, danışman öğretim üyesi olmalıdır.) |
| **I.2.1** | **Adı Soyadı:** [ ] |
| **I.2.2** | **Tarih (gün/ay/yıl):** [ ] |
| **I.2.3** | **İmza:** [ ] |
