# Supplementary material for: Can kinesio tape negatively affect the treatment by creating a hard floor in plantar fasciitis treatment? A randomized clinical trial
Source: PLoS One. 2025 May 5;20(5):e0322397. doi: 10.1371/journal.pone.0322397 (PMC12052111; doi:10.1371/journal.pone.0322397)
Supplement: Attachment — Submitted filename: Response to Reviewers.docx [file pone.0322397.s007.docx]

Dear Editor-in-Chief of,

We have presented the necessary edits regarding our manuscript below.

1. The registration and registration number (**ClinicalTrials.gov**) in the submission data and on the title page of our manuscript is **NCT06516393.**
a) In the methods section of our manuscript, the complete date range for participant recruitment and follow-up is 13/09/2023-18/04/2024. It is located at line numbers 121-122.
b) Our article registration number is NCT06516393. The authors confirm that all ongoing and related trials for this drug/intervention are registered.
2. We have uploaded our trial study protocol and a translation as a supporting information file

Thank you very much for your consideration.

Yours sincerely,

Ass. Prof. Dr. Med. Tuğba KOCAHAN
