# Supplementary material for: Can kinesio tape negatively affect the treatment by creating a hard floor in plantar fasciitis treatment? A randomized clinical trial
Source: PLoS One. 2025 May 5;20(5):e0322397. doi: 10.1371/journal.pone.0322397 (PMC12052111; doi:10.1371/journal.pone.0322397)
Supplement: Attachment — Submitted filename: Response_to_Reviewers_auresp_1.docx [file pone.0322397.s008.docx]

**Comments to the Author:**

**1. Is the manuscript technically sound, and do the data support the conclusions?**

The manuscript must describe a technically sound piece of scientific research with data that supports the conclusions. Experiments must have been conducted rigorously, with appropriate controls, replication, and sample sizes. The conclusions must be drawn appropriately based on the data presented.

Reviewer #1: Yes

Reviewer #2: No

Reviewer #3: No

**Reviewer's Responses to Questions:** Thank you for your comments and suggestions. Revisions have been made in line with your suggestions.

**2. Has the statistical analysis been performed appropriately and rigorously?**

Reviewer #1: I Don't Know

Reviewer #2: No

Reviewer #3: No

Reviewers' comments:

**Reviewer's Responses to Questions:** Statistical analyses were re-performed in line with the suggestions.

**3. Have the authors made all data underlying the findings in their manuscript fully available?**

The PLOS Data policy requires authors to make all data underlying the findings described in their manuscript fully available without restriction, with rare exception (please refer to the Data Availability Statement in the manuscript PDF file). The data should be provided as part of the manuscript or its supporting information, or deposited to a public repository. For example, in addition to summary statistics, the data points behind means, medians and variance measures should be available. If there are restrictions on publicly sharing data—e.g. participant privacy or use of data from a third party—those must be specified.

Reviewer #1: Yes

Reviewer #2: No

Reviewer #3: No

**Reviewer's Responses to Questions:** Data-SPSS file could not be uploaded to the system, but can be sent to the Editor by e-mail if requested.

**4. Is the manuscript presented in an intelligible fashion and written in standard English?**

PLOS ONE does not copyedit accepted manuscripts, so the language in submitted articles must be clear, correct, and unambiguous. Any typographical or grammatical errors should be corrected at revision, so please note any specific errors here.

Reviewer #1: Yes

Reviewer #2: Yes

Reviewer #3: Yes

**Review Comments to the Author:**

**Reviewers' comments#1:** The study from its title is presuming to give a neutral or a known result but it still emphasized if KT and R-ESWT will help in acute care of PF. Systematic reviews and meta- analysis mentioned in the references of the study also suggests that KT or r-ESWT can be aa optional treatment.
Good effort.

**Reviewer's Responses to Questions:** Thank you for your comments.

**Reviewers' comments#2:** A two arm randomized clinical study was conducted which aimed to compare pain scores, Foot Function Index (FFI), activity limitation, gastroc-soleus and plantar fascia flexibility test scores in patients with plantar fasciitis. The conclusions are unclear due to multiple comparison issues.

**Major revisions:**
To avoid issues with multiple comparisons, provide more comprehensive analyses by testing the interaction effect of group by time. If the interaction effect is significant, provide an interpretation of the results, but do not test main effects because the tests for main effects are uninteresting in light of significant interactions. If the interaction effects are non-significant, drop the interaction effects from the model and test the main effects. Determining which results to present when testing interactions is often a multi-step process. A comprehensive reanalysis is required.

**Reviewer's Responses to Questions:**

Thank you for your comments. Main effects and interaction effects were tested. Tests were analyzed using Two Way Analysis of Variance with Repeated Measures for variables normal (Gaussian) distributed. Analyses were performed using Generalized Estimating Equations (GEE) for variables not showing normal distribution. In cases where the interaction term (time*group) was significant, the results were interpreted; results regarding main effects were not taken into account. If the interaction term was not significant, analyses were performed considering the main effects.

**Minor revisions:**
1- In the abstract, briefly identify the statistical methods used to estimate the p-values.

**Reviewer's Responses to Questions:** In the abstract, the statistical methods used to estimate p-values were briefly described; “Two Way Analysis of Variance with Repeated Measures and Generalized Estimating Equations (GEE) methods were used in statistical analyses”

2- Line 123: The power calculation should include: (1) the estimated outcomes in each group; (2) the α (type I) error level; (3) the statistical power (or the β (type II) error level); (4) the target sample size and (5) the statistical testing method and (6) for continuous outcomes, the standard deviation of the measurements. Consider replacing “ideal” with “target” in line 124.

**Reviewer's Responses to Questions:** Thank you for your comments. The corrections you requested have been made; “The α (type I) error level: 0,05; the statistical power (or the β (type II) error level):0,80, the **target** sample size: total 34, each group:17, the statistical testing method: Two Way Analysis of Variance with Repeated Measures, effect size: 0,25 (medium level). The value of 0.25 was determined by expert opinion”.

3- Tables: The columns for the “Test Statistic” can be removed from the tables.

**Reviewer's Responses to Questions:** Test Statistic” columns have been removed from tables.

4- If data is normally distributed, summarize using mean and standard deviation. When the distribution is non-normal, summarize with median, first and third quartiles.

**Reviewer's Responses to Questions:** Thank you for your comments. The corrections you requested have been made.

**Reviewers' comments#3:** While the subject is interesting and potentially insightful for treating plantar fasciitis, the manuscript contains several issues that need to be addressed.

The background is vague and insufficient. Although the abstract is subject to word limitations, the authors have not provided a clear rationale for their work.

The phrase "we believe" is not typically appropriate for use in abstract conclusions and should be revised.

**Reviewer's Responses to Questions:** Thank you for your comments. The background, abstract, and conclusions sections have been revised.

The term "hard floor" is unclear. Kinesio Tape (KT), as an elastic tape, differs from rigid tape in that it does not limit the range of motion, especially with the 25% tension applied, as mentioned in the methods section. More importantly, the term "hard floor," which appears in the title and abstract, is neither explained nor discussed in the text. This needs clarification.

**Reviewer's Responses to Questions:** Thank you for your comments. The term "hard ground" has been clarified.

Lines 57-93 are overly lengthy for the background on plantar fasciitis. These sections should be summarized, retaining only the elements directly relevant to the study. The introduction mentions various factors that can affect plantar fasciitis and its treatment. The methods section should clarify whether all these factors were considered and controlled in the study.

**Reviewer's Responses to Questions:** Thank you for your comments. Lines 57-93 were summarized, leaving only the relevant parts. Inclusion criteria are stated.

The authors refer to "subgrouping the subjects and then randomizing them into one of two groups," which suggests stratified block randomization. This method ensures an equal number of subjects in each group, and the text should explicitly clarify this. Furthermore, several confounding factors were noted in the introduction. It is crucial to specify which of these were controlled in the study groups.

**Reviewer's Responses to Questions:** Thank you for your attention. It was written incorrectly by mistake. This parts has been corrected in the article.

The inclusion criteria lack proper references and should be cited. Similarly, there are no references for the parameters or methods used for r-ESWT, and the flexibility tests also require appropriate citations.

**Reviewer's Responses to Questions:** Thank you for your attention and suggestions. Appropriate references have been added.

It is unusual to leave KT on for one week without changing it. The mechanical properties of KT diminish over time, particularly on a weight-bearing area such as the foot, which is often enclosed in a shoe with socks. Leaving the tape unchanged for a week raises doubts about the validity of the study's results.

**Reviewer's Responses to Questions:** Thank you for your comments. These parts have been corrected in the article. **“**A single tape was applied to the patient's foot for one week. The patient was instructed to remove the tape if it lost its adhesiveness or became loose.” This explanation was added to the text.

The authors need to explicitly mention which variables were normally distributed and specify the tests used for analysis. If the data were normally distributed, a mixed ANOVA (with both within- and between-subject factors) would be the appropriate statistical method.

Since the statistical methods are not clearly explained, the results cannot be thoroughly reviewed. Additionally, effect sizes should be reported alongside p-values.

**Reviewer's Responses to Questions:** Normally (Gaussian) distributed variables were analyzed with Two Way Analysis of Variance with Repeated Measures. Since there are no missing observations in the data, the results of mixed ANOVA and Analysis of Variance with Repeated Measures analysis will be the same. Therefore, mixed ANOVA was not used. Additionally, p-values and effect sizes obtained were reported.

There is a noticeable bias toward r-ESWT throughout the manuscript. Given that there is no significant difference between the two groups for most variables, neither treatment can be considered superior. The conclusion suggesting that KT with r-ESWT could be inferior, as mentioned in lines 276-280, seems biased and is not supported by the data. The authors should discuss the results objectively without bias. Finally, Reference 14 is very similar to the authors' work and should be discussed more thoroughly in both the introduction and discussion sections.

**Reviewer's Responses to Questions:** Thank you for your comments. These parts have been corrected in the article.
